# Macrophyte assessment in European lakes: Diverse approaches but convergent views of ‘good’ ecological status

**DOI:** 10.1016/j.ecolind.2018.06.056

**Published:** 2018-11

**Authors:** Sandra Poikane, Rob Portielje, Luc Denys, Didzis Elferts, Martyn Kelly, Agnieszka Kolada, Helle Mäemets, Geoff Phillips, Martin Søndergaard, Nigel Willby, Marcel S. van den Berg

**Affiliations:** a European Commission Joint Research Centre, Directorate Sustainable Resources, Water and Marine Resources Unit, I-21027 Ispra, VA, Italy; b Rijkswaterstaat Water, Traffic and the Environment, PO Box 2232, 3500 GE Utrecht, The Netherlands; c Research Institute for Nature and Forest (INBO), Havenlaan 88 – 73, 1000 Brussels, Belgium; d Faculty of Biology, University of Latvia, Jelgavas iela 1, Rīga LV1004, Latvia; e Bowburn Consultancy, 11 Monteigne Drive, Bowburn, Durham DH6 5QB, United Kingdom; f Institute of Environmental Protection-National Research Institute, Department of Freshwater Protection, Kolektorska 4, 01-692 Warsaw, Poland; g Estonian University of Life Sciences, Institute of Agricultural and Environmental Sciences, Limnoloogia tee 2/2-3, Rannu, Tartu 61117, Estonia; h Biological and Environmental Sciences, University of Stirling, Stirling FK9 4LA, United Kingdom; i Department of Bioscience, Aarhus University, Vejlsøvej 25, 8600 Silkeborg, Denmark

**Keywords:** Aquatic macrophytes, Ecological status, Eutrophication, Indicator species, Nutrients, Phosphorus, Species richness, Water Framework Directive

## Abstract

The European Water Framework Directive has been adopted by Member States to assess and manage the ecological integrity of surface waters. Specific challenges include harmonizing diverse assessment systems across Europe, linking ecological assessment to restoration measures and reaching a common view on ‘good’ ecological status.

In this study, nine national macrophyte-based approaches for assessing ecological status were compared and harmonized, using a large dataset of 539 European lakes. A macrophyte common metric, representing the average standardized view of each lake by all countries, was used to compare national methods. This was also shown to reflect the total phosphorus (r^2^ = 0.32), total nitrogen (r^2^ = 0.22) as well as chlorophyll-*a* (r^2^ = 0.35–0.38) gradients, providing a link between ecological data, stressors and management decisions. Despite differing assessment approaches and initial differences in classification, a consensus was reached on how type-specific macrophyte assemblages change across the ecological status gradient and where ecological status boundaries should lie.

A marked decline in submerged vegetation, especially Charophyta (characterizing ‘good’ status), and an increase in abundance of free-floating plants (characterizing ‘less than good’ status) were the most significant changes along the ecological status gradient. Macrophyte communities of ‘good’ status lakes were diverse with many charophytes and several *Potamogeton* species. A large number of taxa occurred across the entire gradient, but only a minority dominated at ‘less than good’ status, including filamentous algae, lemnids, nymphaeids, and several elodeids (e.g., *Zannichellia palustris* and *Elodea nuttallii*). Our findings establish a ‘guiding image’ of the macrophyte community at ‘good’ ecological status in hard-water lakes of the Central-Baltic region of Europe.

## Highlights

• We harmonised nine macrophyte-based approaches for assessing lake ecological status.
• We established relationships between the common view and nutrient concentrations.
• Submerged vegetation decrease and free-floating plants increase along the status gradient.
• We describe indicator taxa for ‘good’ and ‘less than good’ ecological status.
• We establish a ‘guiding image’ of the macrophyte community at ‘good’ ecological status.

## Graphical abstract

**Figure:**
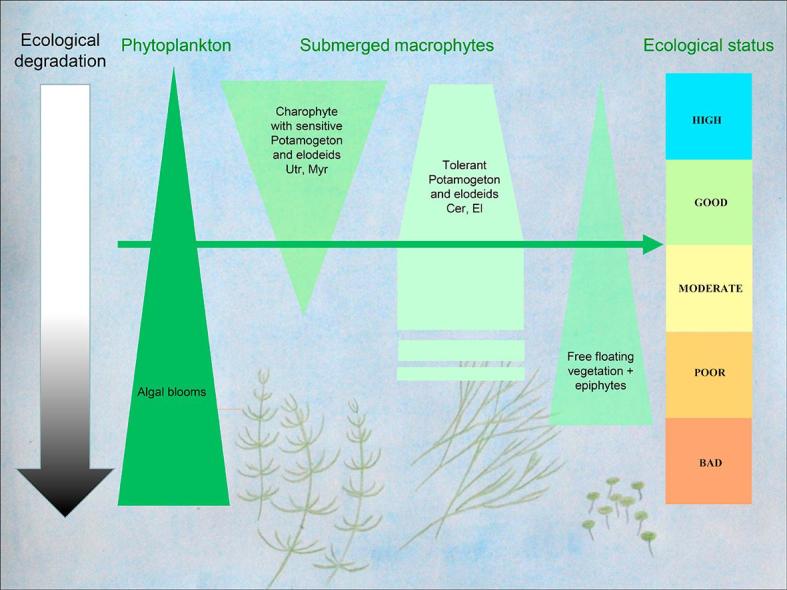

## 1 Introduction

Macrophytes are important components of lake ecosystems, contributing to primary productivity, sediment accumulation and stabilization, storage and cycling of nutrients, as well as providing complex habitat and food for (semi-)aquatic biota from macroinvertebrates to mammals (Jeppesen et al., 2012). In shallow lakes, they are particularly important as they can contribute to a clear-water state through various self-enhancing feedback mechanisms (Scheffer and Carpenter, 2003). Macrophyte communities also contribute to the provision of ecosystem services to society, including sustainable production of food, recreational opportunities, and water purification (Engelhardt and Ritchie, 2001, Hilt et al., 2017).

In most European lakes the composition and abundance of macrophytes has changed because of various human pressures (Körner, 2002, Sand-Jensen et al., 2000). Macrophytes are sensitive to eutrophication (Madgwick et al., 2011, Søndergaard et al., 2010), acidification (Arts, 2002, Brouwer et al., 2002), water level fluctuations (Mjelde et al., 2013, Wantzen et al., 2008), shoreline modifications (Ostendorp et al., 2004), recreation (Asplund and Cook, 1997, Mosisch and Arthington, 2004), navigation (Willby et al., 2001), fish stocking (Williams et al., 2002), and biological invasions (Strayer, 2010).

Many European countries have therefore included macrophytes in their ecological assessment tool-kit, for example, Austria (Pall and Moser, 2009), Denmark (Søndergaard et al., 2010), Germany (Schaumburg et al., 2004), Ireland (Free et al., 2006), Poland (Ciecierska and Kolada, 2014), and UK (Willby et al., 2012). Due to their sedentary nature and relatively slow growth, macrophytes can serve as long-term indicators with high spatial resolution, useful for detecting nutrient enrichment and other impacts occurring at the land–water ecotone (Melzer, 1999, Pall and Moser, 2009).

To ensure comparability of ecological assessment and promote shared levels of ambition among EU member states, the Water Framework Directive (EC, 2000) stipulates that assessment systems are compared and that status boundaries should be adjusted where necessary (Birk et al., 2013). This task of intercalibration has proved challenging, mainly due to intrinsic biogeographical differences between member states and the diversity of sampling, analysis and evaluation approaches they use (Penning et al., 2008a, Poikane et al., 2014b, Poikane et al., 2015). In lowland Europe especially, intercalibration has been hindered by the lack of near-natural reference sites, short pressure gradients, multiple pressures acting on the same sites, confounding factors (e.g. suspended solids and water colour) and different monitoring practices and assessment philosophies (Tóth et al., 2008). In order to overcome these difficulties, innovative approaches have been developed (EC, 2011, Poikane et al., 2014b). A benchmarking procedure allows any typological or biogeographical differences between countries to be removed by normalization (Birk et al., 2013, Poikane et al., 2015). Intercalibration can be carried out by a direct comparison of commonly assessed sites or indirectly, using a common biological or pressure index (Kelly et al., 2014, Poikane et al., 2016a, Poikane et al., 2017). The concept of a ‘harmonisation band’ has been introduced to unify both approaches and to convey the magnitude and direction of deviation of national methods from the global average view of ecological status (Birk et al., 2013).

However, ecological assessment is not a panacea that will single-handedly ensure ‘good’ ecological status of European waters. The next steps toward this challenging goal include diagnosing causes (e.g., nutrient enrichment), defining management targets (e.g., nutrient concentrations) and suggesting restoration measures to remedy the situation. Pressure-response relationships between stressors and biota are one means towards these ends (Karr, 1999, Hering et al., 2010). However, these relationships have not been tested or documented for one-third of the methods proposed so far for the Directive (Birk et al., 2012). Consequently, the necessary links between ecological status and management decisions are obscure or even absent in many river basin management plans, creating one of the most important gaps in the WFD implementation (Hering et al., 2010, Hering et al., 2015).

Last but not least, it is necessary to communicate about the health of lake ecosystems to the public and decision makers (Karr, 1999, Kelly, 2012). Great efforts have been made in ecological assessment to reduce biological communities to metrics and indices. Additional efforts have been made to make these numbers comparable among member states (Birk et al., 2013). Now, it is essential to transform these numbers back into a narrative on healthy aquatic ecosystems and communicate why this is important, not just to ecologists, but also to water managers and citizens (Willby, 2011, Poikane et al., 2016b). A description of biological communities at different ecological status classes (Birk and Willby, 2010) along with a common understanding of community composition at ‘good’ status (‘guiding image’; Palmer et al., 2005) can serve as a first step towards this goal.

In this study, we seek to provide a simple overview of the process of intercalibration performed on assessments of lake ecological status based on macrophytes and then demonstrate that the result has ecological relevance for lake management in Europe.

First, we briefly describe macrophyte assessment methods including the process of intercalibration (i.e. comparison of assessment methods and harmonisation of class boundaries). Next, we establish relationships between the common macrophyte assessment and indicators of the focal pressure, eutrophication. Further, we explore the change of macrophyte communities along the ecological status gradient. Finally, we define those taxa characteristic of ‘high’ and ‘good’ status and contrast them with indicators of ‘less than good’ status. Our findings establish a ‘guiding image’ of the macrophyte community at ‘good’ ecological status in hard-water lakes of the Central-Baltic region of Europe.

## 2 Material and methods

### 2.1 Member state assessment methods

Lake macrophyte assessment methods from nine countries were included in this analysis (Table 1). The macrophyte survey procedures mostly employ transect-based sampling (CEN, 2007), sometimes supplemented by point observations (BE-FL, UK). In all methods hydrophytes (submerged and floating-leaved rooted and non-rooted taxa) are noted, some methods additionally consider helophytes, filamentous algae, mosses and cyanobacterial films (Table 1). Macrophyte abundance is estimated using point- or percentage scales, ranging from 5-point descriptive scale (from very rare = 1 to very frequent = 5) to estimates of percentage coverage on a continuous scale (Table 1).

**Table 1:** Macrophyte methods for lake ecological status assessment included in the analysis; macrophyte groups: SUBM – submerged rooted and non-rooted, FLOAT – floating-leaved rooted and free floating; HELO – helophytes, FILA – filamentous algae (large), MOSS – mosses, CYAN – cyanobacterial films.

Member State	Method and reference	Sampling procedure	Macrophyte groups included	Abundance scale
Belgium Flanders (BE-FL)	Flemish macrophyte assessment system (Schneiders et al., 2004, Leyssen et al., 2005)	Point observations in all homogeneous parts up to 2 or 4 m depth	SUBM, FLOAT, HELO, FILA, MOSS, CYAN	5-point scale for individual taxa and 4-point scale for total abundance

Denmark (DK)	Danish Lake Macrophytes Index (DLMI, Søndergaard et al. 2013)	Transects	SUBM, FLOAT, (FILA)^*^, (MOSS)	6-point scale for each observation point, translated into % coverage

Estonia (EE)	Estonian macrophyte assessment system (Portielje et al., 2014)	Transects for larger lakes, total mapping for smaller	SUBM, FLOAT, (HELO), FILA, MOSS	Separate 5-point scales for SUBM, FLOAT and HELO

Germany (DE)	German Assessment System for Macrophytes & Phytobenthos (Reference Index, Schaumburg et al., 2004)	Transects of ca. 20 m width	SUBM, FLOAT, MOSS	5-point scale for taxa in each depth zone

Latvia (LV)	Latvian macrophyte assessment method (Portielje et al., 2014)	Transects	SUBM, FLOAT, HELO, FILA, MOSS	5- or 7-point scale

Lithuania (LT)	Lithuanian macrophyte assessment method (Portielje et al. 2014)	Transects	SUBM, FLOAT, (HELO), (FILA), MOSS	5-point semi quantitative scale

Netherlands (NL)	WFD-metrics for natural water types (Coops et al., 2007)	Sampling points of a size of 200 × 200 m for larger lakes and transects for smaller lakes	SUBM, FLOAT, HELO, FILA, MOSS	9-point scale for individual taxa and percentage cover for total abundance

Poland (PL)	Ecological Status Macrophyte Index (ESMI, Ciecierska and Kolada, 2014)	Transects of ca. 30 m width	SUBM, FLOAT, HELO, MOSS	7-point scale for taxa and percentage cover for total abundance

United Kingdom (UK)	LEAFPACS lakes macrophyte classification tool (Willby et al., 2012)	Transects of ca. 100 m width	SUBM, FLOAT, FILA, MOSS, CYAN	Percentage cover of each taxa

All assessment systems follow the WFD approach and are thus based on a change from reference condition with status expressed as an Ecological Quality Ratio (EQR), an approach where natural variability is taken into account by assigning lakes to “types”. Therefore, all Member States have developed type-specific reference values that describe the expected value of their index under near-natural conditions for each lake type in their territory. The most common approaches, mostly used in combination, include the use of data from near-natural sites (EE, DE, LT, LV, PL, UK) or historical data (BE, EE, DE, LT, NL, UK); only a few countries used modelling (EE, UK) or palaeolimnological data (DE). All indices are expressed as an EQR ranging from 1 (near-natural conditions) to 0 (heavily impacted) which are divided into five classes of biological quality (High, Good, Moderate, Poor and Bad). Various approaches were adopted to define these ecological boundaries, ranging from uniform division of the EQR scale (BE, NL, PL) to more ecological approaches based on shifts in macrophyte communities, for example, changes in dominance from sensitive to tolerant species (EE, DE, UK).

### 2.2 Lake types and datasets

Biological and environmental data from 539 lakes from nine countries were analyzed. The dataset contained descriptive data (altitude, surface area, mean depth), physical–chemical data (alkalinity, nutrients, chlorophyll-*a*, Secchi depth) as well as macrophyte data. Total phosphorus and chlorophyll-*a* values were averaged over the growing season, which was defined separately by each country to reflect local climate. Macrophyte data were collected by each country using methods shown in Table 1 and reported as species (or higher taxonomic group). For analysis abundance values were transformed into a common categorical scale from 1 to 3 (ECOFRAME scale; Moss et al., 2003).

All the lakes belong to the region covered by the Central-Baltic Geographical Intercalibration Group (Poikāne et al., 2010). Lakes varied significantly in area (0.01–71.4 km^2^) and depth (0.3–15 m), but all were lowland (altitude < 244 m a.s.l.), hard-water (alkalinity > 1.0 meq L^−1^) water bodies. Lakes were allocated to two common intercalibration lake types: LCB1, defined as shallow (mean depth 3–15 m, n = 257) and LCB2, defined as very shallow (mean depth < 3 m, n = 282) (Poikāne et al., 2010).

Lakes represented the entire spectrum of ecological status (macrophyte EQR 0.14–0.90) and trophic conditions, with mean growth season total phosphorus (TP) in the global dataset ranging from 0.006 to 1.46 mg L^−1^, total nitrogen (TN) from 0.03 to 11.9 mg L^−1^, chlorophyll *a* (chl-*a*) from 0.5 to 361.6 µg L^−1^.

### 2.3 Intercalibration methodology

The intercalibration process followed a well-established methodology outlined in the WFD Guidance document (EC, 2011, Birk et al., 2013) and described in detail in Portielje et al., 2014, Poikane et al., 2015. Therefore, only a brief summary is provided below.

Step 1: A common dataset was established and all national methods were applied to the datasets of all other countries (meaning that, for example, each lake of EE was evaluated by assessment systems from the other eight countries);
Step 2: Assessments of lakes by all member states methods were corrected for country effects by continuous benchmark standardization (Birk et al., 2013, Kelly et al., 2014);
Step 3: An ‘Intercalibration Common Metric’ (ICM) was calculated. This is the average EQR of all Member State assessment methods except the native one. Thus, in an exercise involving five countries, for country A the ICM is calculated as the average EQR of countries B, C, D and E; for country B – as the average of countries A, C, D and E, etc.;
Step 4: In order to be retained in the exercise, regression between the ICM and the national EQR should be significant (P < 0.05) with r values > 0.5 and slopes between 0.5 and 1.5 (EC, 2011). If these conditions are not fulfilled, the national method is considered to depart too widely from the common view and has to be modified;
Step 5: The boundary values of the different assessment methods were transformed to the common scale and compared with the global mean view of all countries. National boundaries were adjusted so as not to exceed a deviation of ±0.25 class widths from the global mean of all countries (Birk et al., 2013). This means that the most widely divergent national methods could not differ from each other by more than 0.5 classes in terms of their site classification.

Where low correlations with the common metric were observed and/or where boundaries deviated strongly from the global view of all countries an iterative series of steps were performed to (i) ensure adequate relationship between national method and intercalibration common metric; (ii) to ensure that the boundaries of assessment systems complied with comparability criteria. For several countries (DE, EE, Pl, and UK) class boundaries were adjusted where necessary to ensure compliance with comparability criteria (+/− 0.25 class widths) for HG and GM boundary bias.

### 2.4 Statistical analysis

Statistical analyses were performed using the R software package (R Core Team, 2016).

The response of national macrophyte indices to eutrophication was tested by calculating linear regressions between national indices and water quality indicators (TP, TN and chla-*a*) as well as against average EQR (EQR_avg_). The EQR_avg_ is the average EQR of all intercalibrated assessment methods after benchmark standardization and assumes a consistent view of ecological status between member states.

### 2.5 Analysis of growth forms

Having reduced biological communities to EQRs in order to harmonize status class boundaries, the next step was to translate these values back into meaningful descriptions. This was performed by examining the shift in growth form domination along the eutrophication gradient. Species data were converted to groups differing in growth form (Den Hartog and Van der Velde, 1988). For charophytes, rooted floating-leaved and free-floating vegetation abundance was calculated for individual lake-years by summing up abundance scores for individual taxa.

Abundance of growth forms was analyzed against a common view of ecological status expressed as global mean EQR_avg_ – the average EQR of all intercalibrated assessment methods after benchmark standardisation.

For each plant group and lake type a separate ANOVA was performed using EQR as dependent variable and plant quantity class values as independent values. If there was a significant class effect, Tukey HSD post-hoc test was used to compare classes.

### 2.6 Percentile analysis of indicator taxa

To explore the shift in macrophyte composition with lake degradation, the frequency distribution of taxa over the gradient of the global mean EQR_avg_ was analysed. For all taxa the lowest, 25-percentile, median, 75-percentile, and highest EQR_avg_ of the lake years in which they occurred were calculated. Only taxa that occurred in at least 7 lake-years for either LCB1 or LCB2 from the common database were selected. The frequency of occurrence of taxa was calculated from the number of lake-years with EQR_avg_ at ‘good’ and ‘high’ status (further referred to as ‘good’ status) and EQR_avg_ at ‘moderate’, ‘poor’ and ‘bad’ status (further referred to as ‘less than good status’).

Taxa with more than 75% of their occurrences in lake-years above the ‘good’-‘moderate’ status intercalibration boundary are considered indicative for ‘good’ status. Taxa occurring over the whole EQR_avg_ gradient are considered to be insensitive.

## 3 Results

### 3.1 National macrophyte-based assessment methods

All methods include abundance and compositional metrics; however the designs differ among countries (Table 2). Macrophyte abundance is mostly expressed via colonization depth (DE, DK, EE, LT, and LV), while other countries used cover-related abundance metrics (PL, UK) combined with percentage of different growth forms (BE). Most assessment methods are designed according to the concept of positive, negative and indifferent indicator species (DE, LT, BE, NL) or assign a continuous value to different taxa (UK). Several countries use species or species groups directly in the assessment (DK, LT, and LV). Only a few countries use diversity measures and this is done in different ways – as a number of taxa and functional groups (UK), diversity of growth forms (BE) or syntaxonomic units (PL). Some assessment methods focus purely on eutrophication (e.g. by calibration against total phosphorus concentration: DK, PL, UK), while others focus on more general degradation (BE, NL).

**Table 2:** Metrics included in the macrophyte-based lake assessment systems. ↓ metrics decrease along ecological status gradient; ↑ metrics increase along ecological status gradient.

	Macrophyte state variable		
Member State	Abundance	Structure	Diversity
Belgium Flanders	Area-weighted abundance of submerged vegetation↑↓	Area-weighted type-specific species composition index↓ Area-weighted disturbance index↑	Diversity of macrophyte growth forms↓

Denmark	Maximum colonization depth of submerged macrophytes in deep lakes↓ Coverage of submerged macrophytes (% of total lake area) in shallow lakes↓	Presence of species indicative of nutrient poor conditions↓	

Estonia	Maximum depth of colonization of submerged macrophytes↓	Relative abundance of indicator taxa (*Potamogeton perfoliatius, P. lucens*) or groups (charophytes, bryophytes, lemnids)↓↑ Abundance of large filamentous algae↑	

Germany	Maximum depth of macrophyte stands ↓	Reference Index↓ Dominant stands of the eutrophication indicator taxa↑	

Latvia	Maximum depth of colonization of submerged macrophytes↓	Relative abundance of indicator taxa (*Chara*, *Ceratophyllum* or *Zannichellia*, *Potamogeton perfoliatius*, *P*. *lucens*) or groups (charophytes, bryophytes, lemnids)↓↑ Abundance of large filamentous algae↑	

Lithuania	Maximum depth of macrophyte stands↓	Reference Index↓ Dominant stands of the eutrophication indicator taxa↑	

Netherlands	Relative cover of growth forms↓	Indicator species metrics↓	

Poland	Colonization index (ratio of vegetated area and area where water is shallower than 2.5 m)↓		Pielou’s index (syntax level)↓

UK	Mean percent cover of hydrophytes↓	Lake Macrophyte Nutrient Index (LMNI)↑ Relative cover of filamentous algae↑	Number of hydrophyte taxa↓ Number of functional groups↓

### 3.2 Common view on ecological status and pressure-response relationships

**Fig. 1:**
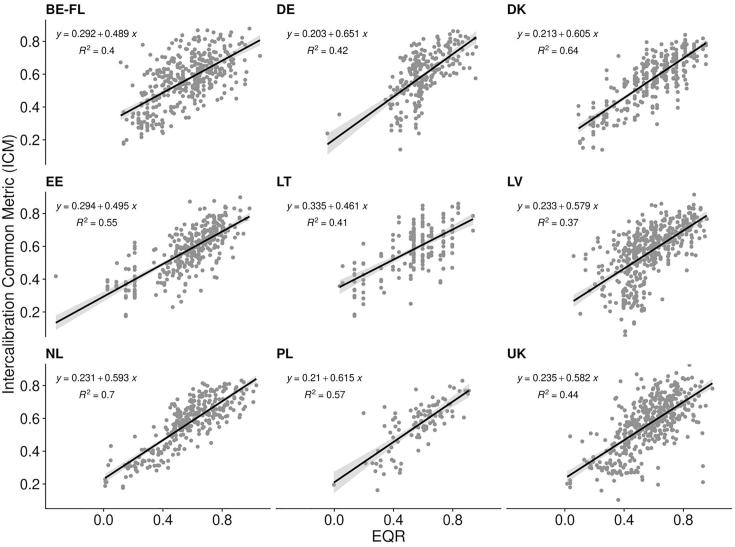
Relationship between member states assessment Ecological Quality Ratios (EQRs) and Intercalibration common metric (ICM), all lakes combined.

After harmonization, Member States assessment indices (expressed as EQR) were strongly and significantly related to the Intercalibration Common Metric (ICM), (Fig. 1, *p* < 0.001; r^2^ ranging from 0.4 to 0.7).

**Fig. 2:**
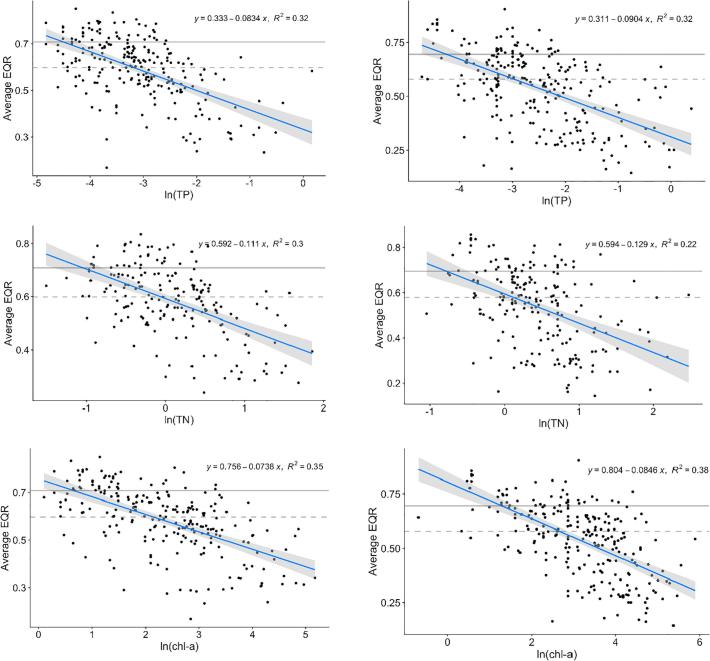
Relationships between log-transformed total phosphorus (TP), chlorophyll-*a* (chl-*a*) and average EQR of all Member States after intercalibration for LCB1 (left) and LCB2 (right). Horizontal lines represent the boundaries of ‘good’ and ‘moderate’ (dashed) or ‘high’ and ‘good’ (solid) ecological status.

The global average macrophyte EQR (average of EQRs of all intercalibrated methods) was also significantly correlated to TP (r = −0.57 both types), TN (r = −0.47 both types) and chl-*a* for (r = −0.59 LCB1 and r = −0.62 LCB2), all significant with P < 0.001 (Fig. 2).

### 3.3 Change of species richness and growth forms along ecological status gradient

**Fig. 3:**
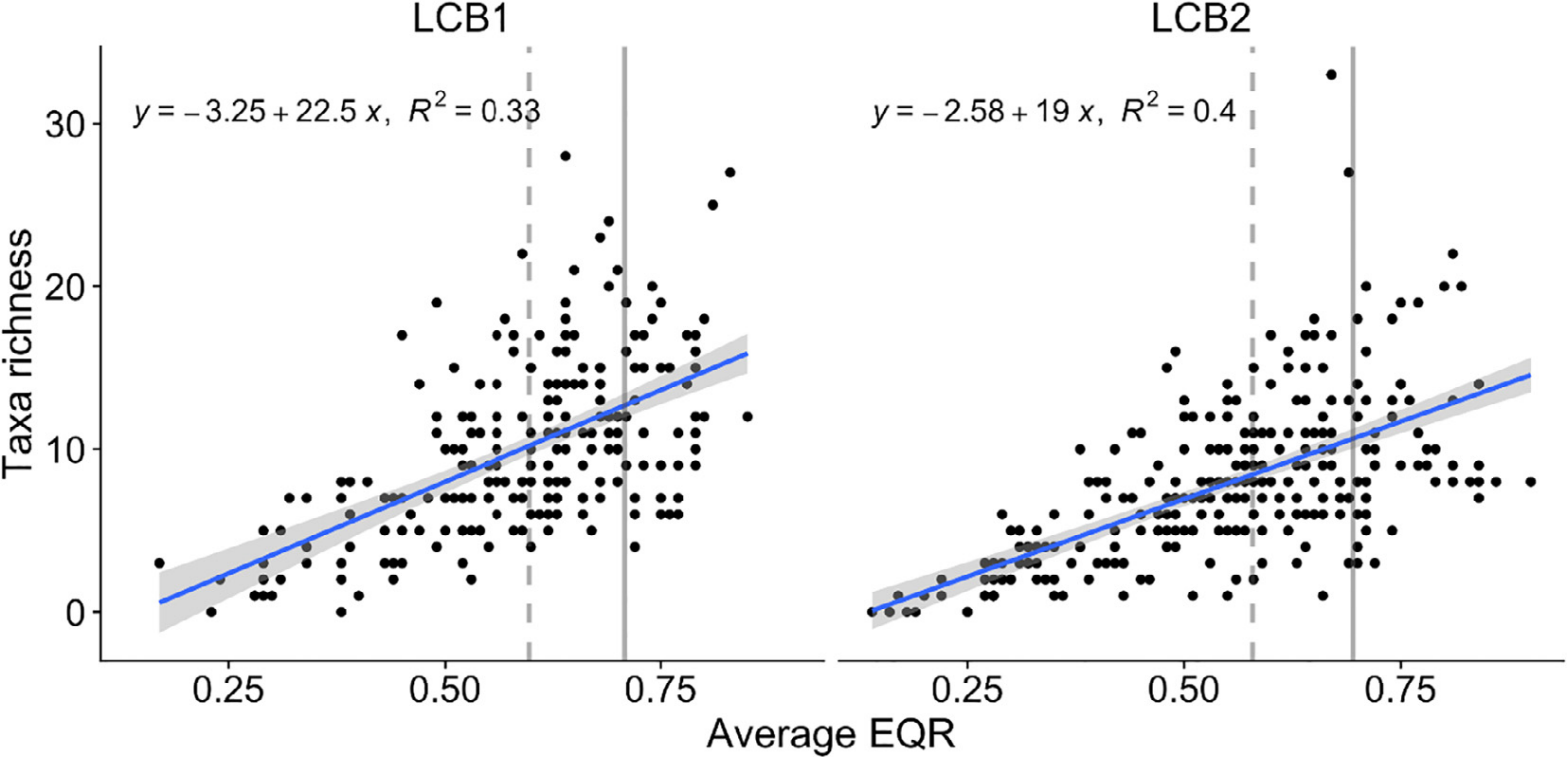
Change of total macrophyte richness along ecological status gradient. Vertical lines represent the boundaries of ‘good’ and ‘moderate’ (dashed) or ‘high’ and ‘good’ (solid) ecological status.

Species richness of aquatic vegetation (Fig. 3) showed an even stronger negative response, with species-rich sites confined exclusively to ‘high’ or ‘good’ status and species-poor sites being found only in the lower status classes.

**Fig. 4:**
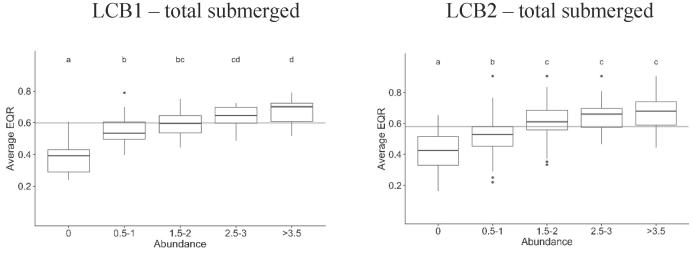
Box plot showing the relationship between EQR_avg_ and total submerged macrophytes in LCB1 and LCB2 lakes. The horizontal line separates ‘good’ and ‘high’ status from ‘less than good’ status. Total submerged macrophyte abundance is calculated in classes ranging from 1 to 5. The macrophyte abundances can be interpreted as follows: submerged macrophyte abundance ≥1.5 – submerged macrophytes are present, at least in low-to-moderate amounts; ≥2.5 – lakes in a macrophyte-dominated state; and ≥3.5 – a high abundance of submerged macrophytes. Different letters indicate abundance classes that are statistically different (p ≤ 0.05).

Submerged macrophytes declined markedly along the ecological status gradient (significant difference among most abundance classes). Lakes without submerged vegetation were associated only with moderate and worse status, whilst lakes with abundant submerged vegetation were associated with ‘good’ and ‘high’ status (Fig. 4).

**Fig. 5:**
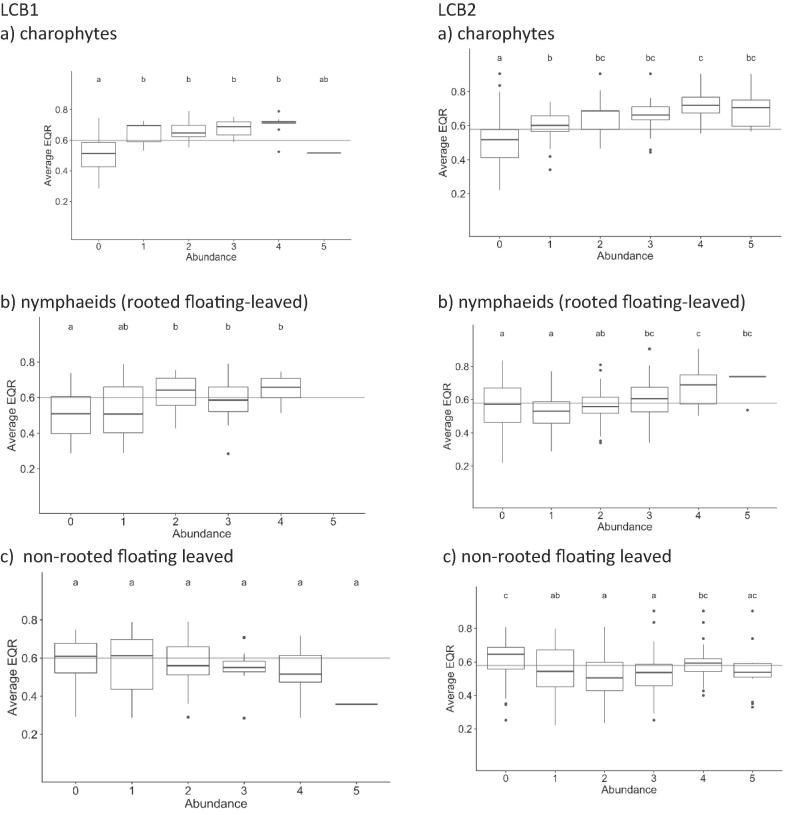
Box plot showing the relationship between EQR_avg_ and growth forms: charophytes (a), nymphaeids (b) and floating plants (c) for LCB1 (left) and LCB2 (right). Different letters indicate abundance classes that are statistically different (p ≤ 0.05). The horizontal line separates ‘good’ and ‘high’ status from ‘less than good’ status.

Charophytes declined along the ecological gradient (Fig. 5a); there was a significant decrease between 0 class (no macrophytes) and other abundance classes, however there were no differences between abundance classes for LCB1, and only class 1 and 4 differed for LCB2 lakes.

For rooted floating-leaved vegetation (Fig. 5b), there was a clear difference between abundance classes 0 and above 2 for LCB1 and 0–1 and above 3 for LCB2, indicating a decline in nymphaeids in the worst part of the ecological status gradient. However, this decline was not as pronounced as for submerged vegetation and thus both charophytes and nymphaeids occur at both good and ‘less than good’ status.

For very shallow lakes (LCB2), the decrease in ecological status was also manifested by increases in non–rooted floating-leaved plants; a high abundance of this group was characteristic for ‘less than good’ status lakes (Fig. 5c). A similar pattern was observed for LCB1 type; however, the change was not statistically significant due to high variability.

### 3.4 Taxa indicating ‘high’, ‘good’ and ‘less than good’ conditions

The list of potential indicator taxa consists of 53 aquatic macrophyte taxa for LCB1 lakes and 50 for LCB2 lakes (only taxa occurring in at least seven lake-years were considered, helophytes excluded). The taxa list was dominated by elodeids (51%) and charids (23%), followed by nymphaeids (12%), lemnids (9%), filamentous algae and bryophytes.

**Fig. 6:**
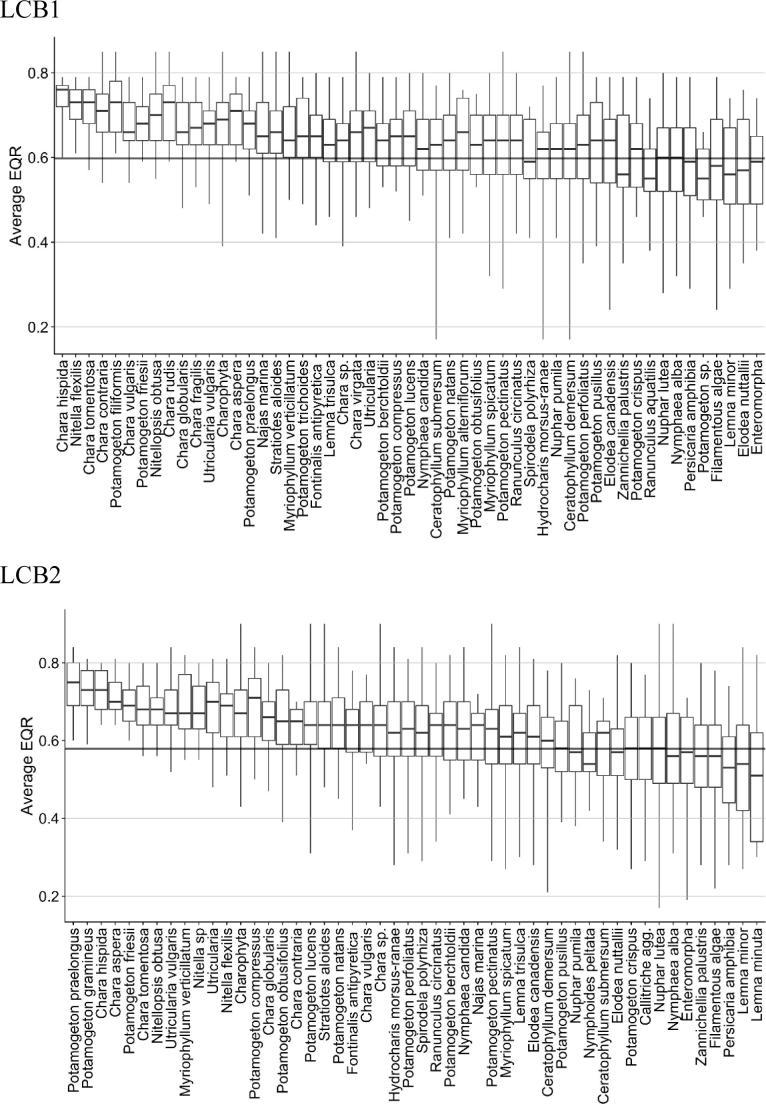
Distribution of macrophyte taxa along the ecological condition gradient (EQR – average value of all MS assessment systems after harmonisation) for LCB1 (above) and LCB2 (below). The horizontal line separates ‘good’ and ‘high’ status from ‘less than good’ status.

Twenty-six taxa (across both lake types) were considered to be characteristic of good status, as these taxa occurred mainly (>75% of observations) in high and good status lakes. Nine taxa for LCB1 type (*Chara contraria*, *C. hispida*, *C. rudis*, *C. tomentosa*, *C. vulgaris*, *Nitella flexilis*, *Nitellopsis obtusa*, *Potamogeton filiformis* and *P. friesii*) and six taxa for LCB2 (*Chara aspera*, *C. hispida*, *Myriophylum verticillatum*, *P. friesii*, *P. gramineus* and *P. praelongus*) can be considered strong indicators, with >90% occurrence in ‘high’ and ‘good’ status (Fig. 6).

Boxplots show 25 and 75% percentiles, medians and ranges of taxa distribution. Sensitive species: 25th percentile > EQR_avg_ for good-moderate status boundary (= 75% occurrence at ‘good’ and ‘high’ status sites).

Good status indicators are taxa that disappear from communities moving down the ecological status gradient. The ‘good’ status indicator list is dominated by charophytes and elodeids (Table 3), several *Potamogeton* species, *Myriophyllum verticillatum*, *Najas marina* (only for LCB1 type), *Stratiotes aloides*, *Utricularia* sp. and *U. vulgaris* (for both types).

**Table 3:** Taxa describing good status for LCB1 and LCB2 lake types. These are taxa with >75% records at good and high status surveys. Br – bryid; Ch – charophyte; El – elodeid; Hy – hydrocharid; Ny – nymphaeid. – = taxa occurring in less than 7 sites (indicator value cannot be determined reliably). N = taxa with <75% records at high and good status (not indicator taxa for this lake type).

Life form	Taxa	Frequency of occurrence at good and high status sites	
		LCB1	LCB2
Ch	*Chara aspera*	0.79	1.0
Ch	*Chara contraria*	0.94	0.85
Ch	*Chara fragilis*	0.88	–
Ch	*Chara globularis*	0.87	0.79
Ch	*Chara hispida*	1.0	1.0
Ch	*Chara rudis*	0.93	–
Ch	*Chara tomentosa*	0.97	0.87
Ch	*Chara vulgaris*	0.90	N
Ch	*Charophyta*	0.88	0.85
Br	*Fontinalis antyipyretica*	0.83	N
El	*Myriophyllum verticillatum*	0.76	0.95
El	*Najas marina*	0.87	N
Ch	*Nitella flexilis*	1.0	0.76
Ch	*Nitella* sp.	–	0.86
Ch	*Nitellopsis obtusa*	0.93	0.87
El	*Potamogeton compressus*	N	0.88
El	*Potamogeton filiformis*	1.0	–
El	*Potamogeton friesii*	0.94	1.0
El	*Potamogeton gramineus*	–	1.0
El	*Potamogeton lucens*	N	0.79
El	*Potamogeton natans*	N	0.76
El	*Potamogeton praelongus*	0.88	1.0
El	*Potamogeton obtusifolius*	N	0.83
Hy	*Stratiotes aloides*	0.82	0.79
El	*Utricularia* sp.	0.75	0.87
El	*Utricularia vulgaris*	0.85	0.82

The majority of the taxa characteristic of good status were the same for LCB1 and LCB2 (charophytes, *P*. *praelongus*, *P. friesii* and *P. gramineus*) but some taxa (e.g. *Najas marina*) were characteristic of good status in only one lake type.

There were no exclusive indicators of high status. This may be due to the relatively small number of lake-years in the database with high status.

The criterion of more than 75% occurrence at ‘less than good’ status could not be applied to identify these indicators, as there were no compliant species. The five species most associated with ‘less than good’ status lakes (Table 4) covered a range of growth forms.

**Table 4:** Taxa associated with ‘moderate and worse’ status for LCB1 and LCB 2 lake types. Al – Algae, El – elodeids, Lm- lemnids, Ny – nymphaeids.

Life form	Taxa	Frequency of occurrence at less than good status sites	
		LCB1	LCB2
Al	Filamentous algae	0.57	0.62
El	*Elodea nuttallii*	0.62	0.56
El	*Zannichellia palustris*	0.63	0.61
Lm	*Lemna minor*	0.60	0.60
Ny	*Persicaria amphibia*	0.52	0.64

Most taxa (∼65%) can thus be considered as indifferent to the EQR gradient and hence for the pressures to which the assessment methods are designed to be responsive. In terms of numbers of indicator species typically present LCB1 lakes supported 4.5 ± 0.2 sensitive species at good or better status compared with 0.9 ± 0.1 in less than good status lakes, while LCB2 lakes supported 4 ± 0.2 sensitive species at good or better status compared with 0.5 ± 0.1 at less than good status.

## 4 Discussion

### 4.1 Macrophyte methods and intercalibration

Macrophyte-based assessment methods have proved indispensable in lake management, especially in lake restoration projects directed at recovery of macrophytes (Coops et al., 2007). However, method development and interpretation of outputs is hampered by the natural spatial and temporal variation inherent in macrophyte communities (Søndergaard et al., 2010) linked to abiotic (e.g. depth, area), biotic (shading by epiphytes, grazing) and stochastic factors (Blindow et al., 1998), as well as uncertainties related to sampling (Kercher et al., 2003). Macrophyte assessment methods have been criticized for their reliance on ‘expert’ judgement, ‘forgotten’ ecology, lack of transparency and lack of well-defined cause-effect-relationships (Demars et al., 2012, Schneider et al., 2016). In reality, such criticisms are generic to any high level ecological assessment that relies on species composition, rather than being specific to macrophytes or freshwater. Additional challenges include the need to harmonise management objectives among European countries given a legacy of disparate monitoring and assessment practices, and the diverse character of high alkalinity lakes (Penning et al., 2008b).

Solutions to these problems can be fostered by compiling and analyzing large transnational databases (Lyche-Solheim et al., 2013), detecting and removing differences between datasets through benchmarking (Birk et al., 2013), describing the change of communities along pressure gradients (Poikane et al., 2014a) and reaching a common understanding on the communities that characterise ‘good’ ecological status (Birk and Willby, 2010).

In our study, we applied these approaches to a large database of 539 lake-years from nine countries to harmonize their macrophyte assessment systems for two common lake types of Central Europe. This was a complex task, in part because of the surprising variety of ways macrophyte communities are measured and assessed among Member states. Considerable differences exist in survey methods, recording of species abundance, inclusion of certain groups (e.g. filamentous algae, bryophytes, helophytes), identification level (e.g. to species, genus or family level for charophytes) and metrics (e.g. diversity indices, abundance indices and sensitivity indices) reflecting different national traditions (Kelly et al., 2015). Do we need so many assessment schemes? Probably not – the harmonization process would have been much easier if monitoring and assessment practices were more closely aligned from the outset. The reasons for such disparity are many, however, and hard to avoid without infringing on the subsidiarity principle.

Nevertheless, a clear common view emerges for LCB1 and LCB2 lakes from the different national assessment methods. Initially, some relatively large disagreements were experienced by the member states but these were resolved through the harmonization exercise. The remaining disagreement largely results from different views on metrics, focus on different pressures and differences in regional species pools and is unlikely to be solved in the near future.

### 4.2 Pressure-response relationships

Pressure-response relationships are a prerequisite for using assessment tools in lake management. Several studies have demonstrated relationships between lake eutrophication and separate macrophyte metrics, for example, colonization depth (Søndergaard et al., 2013), trophic indices (Lyche-Solheim et al., 2013, Penning et al., 2008a), percentage share of *Chara* phytocenoses (Kolada, 2010) and taxa richness (Willby et al., 2012). However, only a few studies demonstrated a strong relationship between overall, holistic, macrophyte assessment and eutrophication. Ciecierska and Kolada (2014), for example, have shown that the Polish index correlates with phosphorus and nitrogen concentrations (r = −0.48 to −0.57) and Free et al. (2006) demonstrated how the Irish index responded to total phosphorus (r = −0.77).

In our study, we established relationships between individual national assessments and the average of all national assessments on one hand and to eutrophication pressure, expressed as TP, TN and chlorophyll-*a*, on the other. The correlation coefficients between national methods and the pressure indicators ranged from −0.3 to −0.7 and are relatively low compared to that between phytoplankton and nutrients (Phillips et al., 2013). However, the relationships with average assessments (r = −0.57 for TP, to −0.47 for TN and −0.59 to −0.62 for chl-*a*) are significant and similar to those recorded in other studies (Birk et al., 2012).

Firstly, high variability in pressure-response relations of macrophytes to eutrophication arises from their sensitivity to pressures other than eutrophication, for example, water-level changes (Mjelde et al., 2013, Penning et al., 2008a). Even eutrophication is reflected only partly by key variables measured in the water column (Schneider et al., 2016), whilst macrophytes also depend on sediment quality and nutrient concentrations (Verhofstad et al., 2017). Secondly, and in contrast to phytoplankton, the response of macrophytes to nutrients appears to be mostly indirect, arising from reduced transparency due to shading by phytoplankton and epiphytes, and biotic interactions (Scheffer and van Nes, 2007). Thirdly, macrophytes are influenced by many natural factors, including, lake area and altitude (Rørslett, 1991), climatic conditions (Rooney and Kalff, 2000, Scheffer and van Nes, 2007), and wind exposure (Feldmann and Nõges, 2007, Spence, 1982). Finally, variability was magnified by applying national assessment systems to data of countries for which these systems were not specifically calibrated.

Striving for strong pressure-response relationships has been advocated by several authors, for instance Prairie (1996) who suggested a threshold r^2^ ≥ 0.65 (see also Bryhn and Dimberg, 2011). However, such strong relationships may be often unrealistic in aquatic sciences, considering that even the seemingly straightforward relationship between chlorophyll-*a* and total phosphorus fails to meet this criterion (Lyche-Solheim et al., 2013, Phillips et al., 2013). Moreover, ‘overly’ strong relationships could ultimately render biology redundant in the classification process (Willby et al., 2012).

### 4.3 Response of macrophyte growth forms

Our study shows a marked decline of submerged vegetation along the ecological status gradient, consistent with numerous other studies (Coops et al., 2007, Egertson et al., 2004, Han and Cui, 2016, Hilt et al., 2013, Poikane et al., 2014a, Sand-Jensen et al., 2000, Sand-Jensen et al., 2008, Søndergaard et al., 2010).

According to our data, the most characteristic feature of ‘good status’ is the presence of charophytes, which were commonly absent from ‘less than good’ lakes. Many studies have linked charophytes with low nutrient concentrations and a clear water state (Scheffer and van Nes, 2007). However, some studies have noted high abundance of *Chara* at quite high nutrient concentrations (del Pozo et al., 2010, Søndergaard et al., 2010) and therefore questioned their indicator value (Søndergaard et al., 2010). This reflects the self-stabilising property of charophyte meadows, maintaining a clear water state also at relatively high nutrient levels (Scheffer and van Nes, 2007).

In contrast, rooted floating-leaved plants occurred across the complete ecological status gradient, albeit decreasing significantly below good status. This conflicts with the reported positive response of floating-leaved plants to nutrients. In theory, a shift from low-growing macrophytes, such as charophytes, to canopy-forming and floating-leaved species occurs as light becomes limiting for submerged vegetation (Moss et al., 2003). However, most empirical studies little response of floating-leaved species abundance (Kolada, 2010), richness (Jeppesen et al., 2000) or both (del Pozo et al., 2010, Han and Cui, 2016) to eutrophication pressure indicators. Moreover, del Pozo et al. (2010) describe a substantial decline in floating-leaved richness and abundance along a combined pressure gradient including shore alteration, livestock use and dredging, with the best ponds showing 40% floating-leaved cover compared to none in the most impacted ponds. The likely explanation is that floating-leaved plants are quite tolerant to eutrophication (due to their insensitivity to shading by phytoplankton and epiphytes), only disappearing in the final stages, but are, however, susceptible to other pressures, especially hydromorphological alterations or management. For instance, Mjelde et al. (2013) illustrate the sensitivity of floating-leaved taxa to water-level fluctuations in Nordic lakes. We could not test this hypothesis, as Europe-wide data on hydromorphological pressures are scarce. However, the need to better understand these impacts is a priority for future research (Reyjol et al., 2014).

In contrast, free-floating plants showed a clear increase as ecological status deteriorated, in line with other studies (Portielje and Roijackers, 1995, Vaithiyanathan and Richardson, 1999) and consistent with their growth strategy (Scheffer and van Nes, 2007), being unrestricted by the underwater light climate and benefiting from increased nutrient availability in the water column.

### 4.4 Indicator taxa

The indicator species concept (Carignan and Villard, 2002) is widely used in lake and river assessment across a range of biota. Several approaches are used to define indicator species: (1) expert judgement in combination with evidence from the literature (Melzer, 1999); (2) characterising communities of pre-selected group, e.g., reference lakes (Järvinen et al., 2013) (3) relating species occurrence to pressures, usually (in the case of photosynthetic organisms) total phosphorus concentration or, in some cases, chlorophyll-*a* (Schneider and Melzer, 2003, Willby et al., 2012). For instance, Søndergaard et al. (2010) defined nutrient-poor indicator species as those for whom 75% of observations were from lakes with chl-*a* <25 µg L^−1^ and TP < 0.05 mg L^−1^. Penning et al. (2008b) defined sensitive species as having 75% of their occurrences in lakes with TP < 0.06 mg L^−1^ (for high alkalinity lakes in the Central-Baltic region) or <0.03 mg L^−1^ (all lakes in Nordic region, except Norway). In our study, we used a similar principle, with an important difference in that species are linked not to nutrient concentrations, but to an ecological status assessment harmonised among member states. Therefore, the selected taxa reflect the common view of member states on what macrophyte communities in ‘good’ status lakes should include.

Twenty-six species were indicative of good status for hard-water lakes of Europe, including charophytes (*Chara*, *Nitella*, *Nitellopsis*), several *Potamogeton* and elodeid species. The dominance of charophytes, their role in maintaining a clear-water state, and their decline due to lake degradation is well described and understood (Blindow et al., 2014, Scheffer and van Nes, 2007, Van den Berg et al., 1999).

The indicator value of *Potamogeton* spp. is much less clear. Some studies treat *Potamogeton* as a group (Kolada, 2010) despite differences in growth form and strategy, while others point out that high heterogeneity within this group hampers their use in lake assessment (Han and Cui, 2016). Our study suggests that there are at least two different response groups in Potamogetonaceae, one comprising species typically associated with clear water conditions: *Potamogeton compressus*, *P. filiformis*, *P. friesii*, *P. gramineus* and *P. praelongus*. The widespread disappearance of these taxa is reported for lakes and rivers in Denmark (Riis and Sand-Jensen, 2001, Sand-Jensen et al., 2000, Sand-Jensen et al., 2008), lakes in Finland (Rintanen, 1996) and Iowa, US (Egertson et al., 2004) and lowland freshwaters in the UK (Preston and Croft, 1997). The second main group is broadly disturbance-tolerant and commonly persists in shallow eutrophic lakes: *Potamogeton crispus*, *P. pectinatus*, *P. perfoliatus*, and *P. pusillus*. These differences are linked to individual growth strategies: clear-water *Potamogeton* mostly represent slower-growing species with low expansion capacity, susceptible to turbidity and competition from large angiosperms, whereas tolerant *Potamogeton* species are fast-growing nutrient-demanding species capable of forming a dense canopy at or just below the water surface. For instance, *P. pectinatus* relies on energy from tubers in early summer to rapidly extend shoots and overcome light limitation (van Wijk, 1988). Two species, *Potamogeton natans* and *P. lucens* may represent a third transitional group. While the former is widespread, the latter has declined in lowland freshwaters in north-west Europe (Sand-Jensen et al., 2000, Preston and Croft, 1997) and would generally therefore be considered a desirable species (Willby et al., 2012), yet in the Baltic states of Estonia and Lithuania *P. natans* is regarded as an indicator of poor status (Portielje et al., 2014). Conflicting views on the indicator values may reflect the fact that some species benefit from low levels of enrichment, thus reflecting small differences between countries in their baseline conditions or length of available gradient of ecological quality.

Several contrasting *elodeid* species also characterise ‘good’ status: *Myriophyllum verticillatum*, *Najas marina* (only LCB1 lakes) and *Utricularia vulgaris* (both types), as well as *Stratiotes aloides* (both types). Elodeids are typically regarded as eutrophication indicators (Moss et al., 2003) yet several studies report the decline of *Utricularia* sp. (Sand-Jensen et al., 2008, Vaithiyanathan and Richardson, 1999) and *Myriophyllum* sp. (Hilt et al., 2013, Rintanen, 1996, Sayer et al., 2010) with eutrophication. *M. verticillatum* and *U. australis* are listed as taxa indicative of nutrient-poor conditions in Denmark (Portielje et al., 2014) and *U. vulgaris* as such in Ireland (Free et al., 2006). *Stratiotes aloides* and *Utricularia vulgaris* are included as “reference taxa” in the German assessment system (Portielje et al., 2014). In the UK *Myriophyllum verticillatum, Najas marina* and *Utricularia vulgaris* are considered to characterise ‘high’ status hard-water lakes (Willby et al., 2012), a view consistent with palaeolimnological evidence (Madgwick et al., 2011).

No species met the equivalent threshold (75% occurrence) for defining ‘less than good’ status, in part because poorer status was associated with a reduction in macrophyte abundance and richness. The few taxa characteristic of ‘less than good’ status (Table 4) react positively to eutrophication and thrive with a high nutrient supply but often also co-occur with sensitive taxa at higher ecological status. The ‘less than good’ status indicators include lemnids (*Lemna minor*) and nymphaeids (*Persicaria amphibia*) which tolerate high turbidity by concentrating their biomass at the water surface during summer. Filamentous algae were also indicators of ‘less than good’ status – several studies have shown positive associations between their development and nutrient enrichment (del Pozo et al., 2010, Willby et al., 2012), in spite of the taxonomic (and ecological) heterogeneity within filamentous algae, and epiphytic algae are considered key mediators in the switch to phytoplankton dominance in eutrophic lakes (Phillips et al., 2013). Several elodeids – *Zannichellia palustris*, *Elodea nuttalli –* also characterise ‘less than good’ status. *Zannichellia palustris* is commonly associated with nutrient-rich conditions (Melzer, 1999, Penning et al., 2008b, Sayer et al., 2010, Søndergaard et al., 2010,) and the invasive *Elodea nuttallii* is considered a nutrient-demanding and disturbance-tolerant species with high dispersal capacity (Zehnsdorf et al., 2015), typical of lakes with high nutrient levels (Coops et al., 2007, Søndergaard et al., 2010).

### 4.5 A ‘guiding image’ of good ecological status

There is an extensive body of work on how macrophyte communities respond to anthropogenic pressures that underpins macrophyte-based assessment methods (Penning et al., 2008a, Penning et al., 2008b, Søndergaard et al., 2010, Søndergaard et al., 2013). However, surprisingly few studies deal with the question of defining good status for the macrophyte assemblage. This is rather fundamental, as it decides management objectives and restoration actions to reach these objectives, and, in the end, shapes the future of European freshwaters.

It is widely accepted that target setting should be based on pressure-response relationships (Karr, 1999, Lyche-Solheim et al., 2013) and that thresholds should play a key role in boundary setting (Poikane et al., 2014a). It is also important to include societal values in the development of indices and class boundaries (Suplee et al., 2009). However, the practice is somewhat different where arbitrary equidistant division of the EQR scale is adopted for boundary setting (Birk et al., 2012, Ciecierska and Kolada, 2014). One consequence of this approach is that it becomes difficult to convey the meaning of ‘good’ status in its broadest sense to scientists and to the public and decision makers (Kelly, 2012, Poikane et al., 2016b, Willby, 2011).

**Fig. 7:**
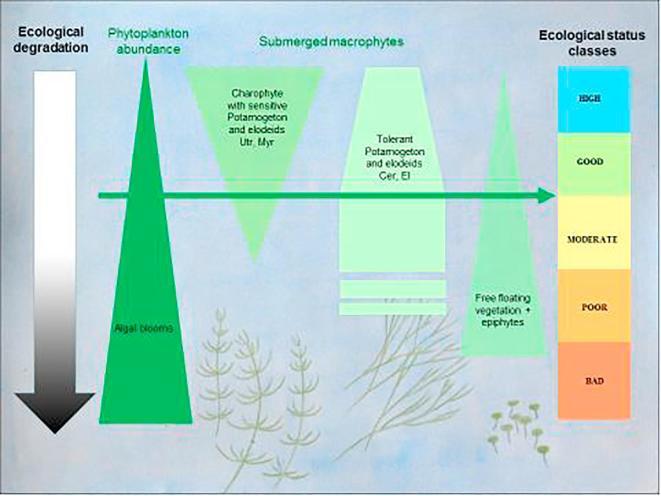
Shift of the macrophyte community along the ecological status gradient and emerging perception of ‘good’ ecological status for European hard-water lakes.

Nine countries independently developed their view of ‘good’ ecological status, which was further harmonised and tested with a common dataset. Despite the high variability introduced by local environmental factors and different national designs and practices, a common view emerged of the change in macrophyte communities with human pressure. Thus, along the ecological status gradient a shift occurs from dominance of charophytes to tolerant and canopy-forming elodeids, followed by disappearance of submerged vegetation and expansion of free-floating vegetation. The key to ‘good’ ecological status, therefore, is that a lake must remain dominated by diverse stands of submerged vegetation consisting of charophyta, sensitive *Potamogeton* spp. and several other taxa (*Myriophyllum* sp., *Utricularia* sp.) (Fig. 7). In the next stage, with the loss of sensitive species, submerged vegetation is dominated by species-poor stands of tolerant elodeids, while in the final stage submerged vegetation disappears. It is important to stress that in soft-water lakes the pattern would be different, involving disappearance of the isoetid community (Penning et al., 2008b).

What are the benefits of setting a ‘guiding image’ for ‘good’ ecological status? Firstly, it helps to convey ecological data to the public and non-technical stakeholders in a more visual way (Suplee et al., 2009, Willby, 2011). Secondly, it supports to link ecosystem status to functions and ecosystem services (Hilt et al., 2017). Last, it helps to guide restoration measures towards a shared vision against which progress can be measured (Palmer et al., 2005). Ultimately, it helps to create a common view on targets and measures on a European scale, while taking into account geographical differences.
